# Recent developments in evaluation and treatment of lateral patellar instability

**DOI:** 10.1186/s40634-017-0119-z

**Published:** 2018-01-10

**Authors:** Alexander Zimmerer, Christian Sobau, Peter Balcarek

**Affiliations:** Arcus Sportklinik, Pforzheim, Germany

**Keywords:** Patellar instability, First-time dislocation, Trochlear dysplasia, Trochleoplasty, Torsion, Rehabilitation, Outcome

## Abstract

Recent years have been characterized by an ongoing increase in knowledge about the different conditions associated with lateral patellar instability. This increase in knowledge provides differentiated approaches to the various pathologies of the patellofemoral joint. Though current guidelines consider medial patellofemoral ligament (MPFL) reconstruction the basic treatment for the unstable patella, medial soft tissue–stabilizing procedures should not be interpreted as stand-alone procedures in every case. The influence of different anatomical factors leading to patellar instability, as well as their impact on clinical outcome measures, is becoming increasingly apparent and deserves further attention. Therefore, the purpose of this review was to summarize recent developments in lateral patellar instability beyond MPFL reconstruction techniques. For this goal, the literature published within the last 3 years considering all aspects of lateral patellar instability was analysed. Six main topics evolved according to the number of publications and in terms of novel aspects and recent developments in the evaluation and treatment of lateral patellar instability. Those topics formed the basis of this article: (1) treatment of first-time patellar dislocation, (2) the impact of trochlear dysplasia and trochleoplasty procedures, (3) the relevance of torsional deformities, (4) patellar instability in open physis, (5) the implementation of new outcome measures, and (6) rehabilitation after patellar stabilizing procedures.

## Background

The discovery of the medial patellofemoral ligament (MPFL) and the development of MPFL reconstruction techniques was the decisive milestone in the treatment of lateral patellar instability within the last 10–15 years. Meanwhile, many different MPFL reconstruction techniques have been developed, which, if carried out correctly, provide good to excellent clinical results with low redislocation rates. For this reason, current guidelines consider MPFL reconstruction to be the basic treatment for lateral patellar instability (Liebensteiner et al. 2017). MPFL reconstruction or other medial soft tissue–stabilizing procedures, however, should not be interpreted as stand-alone procedures in every case. The influence of different anatomical factors leading to patellar instability, as well as their effect on clinical outcome measures, is becoming increasingly apparent and deserves further attention. In addition, treatment of first-time patellar dislocation is still controversial, and the youngest patients with open physis face particular challenges. A glance at the current developments in patellar instability beyond novelties in MPFL reconstruction appears meaningful and necessary and was the objective of this review article.

## Material and methods

MEDLINE was searched using the following algorithm: ((“patella”[MeSH Terms] OR “patella”[All Fields] OR “patellar”[All Fields]) AND instability[All Fields]) OR (“patellar dislocation”[MeSH Terms] OR (“patellar”[All Fields] AND “dislocation”[All Fields]) OR “patellar dislocation”[All Fields]). Titles and abstracts of all articles published between January 1, 2014, and July 31, 2017, were screened for potential new aspects in evaluation and treatment of lateral patellar instability. Exclusion criteria were case-reports, articles published in a language other than English or German, articles related to total knee arthroplasty, medial (iatrogenic) patellar instability, and studies from veterinary medicine.

Abstracts of those titles that were considered potentially relevant for this review were further evaluated for eligibility. Afterwards, selected articles underwent full-text analysis, and the main topics were defined. In addition, a cross-reference search was carried out for the same publication period.

## Results

The initial search algorithm identified 776 articles. According to the exclusion criteria, 210 articles were rejected. During the evaluation process of the 566 remaining titles and abstracts, six main topics evolved according to the number of publications that discussed them and in terms of novel aspects and recent developments in evaluation and treatment of lateral patellar instability. Those topics formed the basis of this article (Table 1).

**Table 1:** Selected main subjects containg potential new aspects of patellar instability with number of publications (January 2014 to August 2017)

Main topic	Number of publications
Treatment of first-time patellar dislocation	18
Trochlear dysplasia and Trochleoplasty	25
Torsional deformity	16
Scoring of patellofemoral disorders	8
Patellar instability in children and adolescents	31
Rehabilitation after patellar stabilization	14

Although new aspects were also recognized in some other areas, including the assessment of the tibial tuberosity–trochlear groove (TTTG) distance and tibial tuberosity–posterior cruciate ligament (TTPCL) distance, new anatomical aspects of the secondary medial passive restraints, i.e., the medial patellotibial and patellomeniscal ligament, and the use of artificial ligaments for MPFL reconstruction, those were not included in this review.

### Treatment of first-time patellar dislocation

Current literature suggests that those patients with a high risk of recurrent patellofemoral instability after an initial dislocating event may benefit from surgical treatment (Nwachukwu et al. 2016).

A recent published study reported an overall annual incidence of acute lateral patellar dislocation of about 23.2 per 100.000 person-years (Sanders et al. 2017). Especially among adolescents between 14 and 18 years, the incidence was highest, at 147.7 per 100.000 person-years, which was greater than previously reported and showed no difference between the sexes (Sanders et al. 2017).

Based on the outcomes after medial soft-tissue reefing and lateral release procedures, non-operative treatment was still the leading recommendation after primary patellar dislocation until the last 4 to 5 years, except in the presence of an osteochondral flake fracture (Stefancin and Parker 2007; Smith et al. 2011; Zheng et al. 2014). However, patellar redislocation occurs in 15% to 72% of patients treated non-operatively, and even patients who did not report recurrent patellar instability episodes following non-operative treatment often did not restore their pre-injury function and reported persistent limitations in their daily life activities within 3 years after the primary dislocating event (Magnussen et al. 2017). In addition, recent studies have shown a high risk of patellofemoral arthritis after non-operative treatment of lateral patellar dislocation in the long term (Sanders et al. 2017; Salonen et al. 2017).

Recent studies now seem to contradict the dogma of conservative treatment in primary patellar dislocations, particularly since the MPFL reconstruction technique has undergone a more widespread implementation in the treatment of first-time patellar instability (Bitar et al. 2015; Bitar et al. 2012). Several meta-analyses reported significant reductions of patellar redislocation rates after surgical treatment and improved Hughston VAS when compared to non-operatively treated patients (Erickson et al. 2015; Saccomanno et al. 2016; Khan and Miller 2016; Wang et al. 2016; Smith et al. 2015). Therefore, a paradigm shift has occurred from conservative to primary operative treatment of first-time dislocations. However, reduced redislocation rates are so far not necessarily reflected in the clinical results, at least not when the Kujala score is used as the clinical outcome measure (Wang et al. 2016; Saccomanno et al. 2016; Erickson et al. 2015), a limitation that will be discussed later. It is therefore difficult to make a general recommendation on surgical therapy for first-time patellar dislocation at this time. Currently, an individual clinical decision-making approach has been favoured, taking each individual risk factor of patellar redislocation into account (Liebensteiner et al. 2017; Jaquith and Parikh 2015). To define objective criteria to identify patients with a higher risk of patellar redislocation, Balcarek et al. (2014) introduced the Patellar Instability Severity Score, which includes six factors (age, bilateral instability, patella alta, TT-TG distance, patellar tilt and trochlear dysplasia) with a maximum score of 7 points. Patients who scored ≥ 4 points showed a nearly 5-times higher redislocation rate than patients who scored ≤ 3 points (Balcarek et al. 2014). Similarly, Jaquith and Parikh (2015) showed that the risk of a patellar redislocation increased with the number of underlying risk factors. Factors include trochlear dysplasia, skeletal immaturity, patella alta, and history of a contralateral dislocation. If all four factors were present, the estimated risk of a patellar redislocation increased up to 88% (Jaquith and Parikh 2015).

### Trochlear dysplasia and Trochleoplasty

In the 1990s, H. Dejour and colleagues established the dysplastic trochlear groove as the most common predisposing factor of patellar instability and current literature increasingly supports trochleoplasty procedures for the treatment of lateral patellar dislocations.

The biomechanical impact of trochlear dysplasia was confirmed by several cadaver studies and computational analyses, which identified the sulcus angle as the most impactful part of total patellofemoral constraint (van Haver et al. 2015; Fitzpatrick et al. 2016). In addition, a clear correlation between trochlear dysplasia and the development of cartilage defects and osteoarthritis in the patellofemoral joint has been established (Sanders et al. 2017). In particular, the elevated antero-lateral femoral cortex, i.e., the trochlear ‘bump’ or ‘spur’ as seen in severe trochlear dysplasia types B and D according to Dejour, results in a reduced patellofemoral contact area, an increased contact pressure, and an increased lateralization and translation of the patella (van Haver et al. 2015). The removal of this femoral prominence with remodelling of the trochlea to a physiological shape is therefore the rationale for carrying out a deepening trochleoplasty. Despite the clear biomechanical parameters, it took a long time to make trochleoplasty an established procedure in the multimodal treatment concept of lateral patellar instability. Concerned about the potential risk of late patellofemoral osteoarthritis, many authors have considered the trochleoplasty procedure primarily a good salvage option for patients with unsuccessful previous surgery, in which the severely dysplastic trochlea remains the main pathologic factor (Balcarek et al. 2016). However, recent studies showed an increasing correlation between the severity of trochlear dysplasia, the clinical outcome, and the redislocation rate after isolated soft-tissue patellar-stabilizing procedures, even after MPFL reconstruction in patients with underlying severe trochlear dysplasia (Kita et al. 2015; Wagner et al. 2013; Hiemstra et al. 2016; Hiemstra et al. 2017). Particularly, in severe dysplastic trochlea, Balcarek et al. (2016) found that the likelihood of protecting the patella from subsequent post-operative redislocation/subluxation was greater in patients who underwent a trochleoplasty plus individual extensor apparatus balancing compared to MPFL reconstruction alone (Balcarek et al. 2016). Thus, trochleoplasty should be increasingly considered a primary treatment option. In addition, the current literature remains inconclusive regarding any link between trochleoplasty and patellofemoral osteoarthritis (Song et al. 2014). In contrast, trochleoplasty seems to have a protective effect for the patellofemoral joint by normalizing patellofemoral kinematics and contact pressures, but this effect continues to be investigated. However, it seems clear that cartilage therapy in a dysplastic trochlea can hardly work. Overall, all three deepening trochleoplasty techniques (the Bereiter “U-shaped” trochleoplasty, the Dejour “V-shaped” trochleoplasty, and Goutallier’s “recession trochleoplasty”) showed significantly improved stability and function, relatively low rates of osteoarthritis and pain, and a moderate rate of complications (Longo et al. 2017).

In clinical practice, a reliable and valid classification of the dysplastic trochlea remains a major challenge. To improve observer agreement, it has been recommended that the four-part Dejour classification (A-D) should be grouped only into low-grade (Type A) and high-grades (Types B-D). The situation is made more difficult by the fact that quantitative single-measurement parameters of the femoral trochlea, i.e., the sulcus angle, trochlear depth, or trochlear facet asymmetry, are of limited value for the assessment of the complex three-dimensional trochlear anatomy (Nelitz et al. 2014), though the lateral trochlea inclination angle was rated as the most appropriate measure by an expert panel (Paiva et al. 2017). In addition, Tscholl et al. (2017) showed that trochlear dysplasia measured on lateral radiographs and MRI showed only fair agreement, especially when the supratrochlear region of the distal femur was not analysed, and MRI analysis that considered only the cartilaginous trochlea tended to underestimate the severity of dysplasia (Tscholl et al. 2017). For a more precise evaluation, they recommended analysing the entire distal femur on axial MRI (Tscholl et al. 2017). For the assessment and clinical decision-making for or against a trochleoplasty procedure, it appears mandatory to look closely at the clinical situation of each patient (for example: positive j-sign, instability or positive apprehension test > 30° of knee joint flexion), and to assess more than one parameter of trochlear dysplasia using both true-lateral radiographs and axial MRI images.

### Torsional deformity

Although a torsional deformity was considered a risk factor for patellar instability in the 1980s and 1990s, little attention was paid to femur antetorsion (anteversion) and external tibia torsion within the portfolio of patellar instability until a few years ago. Nowadays, femur antetorsion in particular is relevant in patellar instability and anterior knee pain.

In a recent biomechanical study, Kaiser et al. (2017) showed that in the MPFL-intact state 20° of increased femur antetorsion significantly increased the force shift of the patella towards the lateral side, whereas in the MPFL-deficient knees, merely 10° of increased femur antetorsion represented a significant risk factor for patellar instability (Kaiser et al. 2017), a constitution typically seen in nearly all cases after recurrent patellar dislocation. Fitzpatrick et al. (2016), using a computational finite element analysis to study multiple factors contributing to patellar instability, found that the antetorsion of the femur contributed about 10–15% to the overall constraint of the patellofemoral joint (Fitzpatrick et al. 2016). This relationship has also been confirmed by recent clinical studies. Increased femur antetorsion in patients with recurrent patellar dislocations had a negative effect on the outcome of anteromedialization of the tibial tuberosity combined with MPFL reconstruction (Franciozi et al. 2017). Similarly, Nelitz et al. (2014, 2014) identified the ignored high femur antetorsion as a reason for revision surgery after MPFL reconstruction (Nelitz et al. 2014). Other studies described good results after a derotating (torsional) osteotomy of the femur or tibia in selected patients, in which the symptoms of anterior knee pain or patella instability were mainly triggered by a torsional deformity (Nelitz et al. 2015; Dickschas et al. 2017; Drexler et al. 2014; Dickschas et al. 2015; Stevens et al. 2014). However, there is no clear consensus regarding the normal limits or the torsion threshold to indicate surgical correction by derotational osteotomy. Within published literature, the mean extent of torsional correction ranged from 11° to 25° for the femur and from 11° to 36° for the tibia (Dickschas et al. 2015; Dickschas et al. 2017; Drexler et al. 2014; Stevens et al. 2014). Based on these data, a derotational osteotomy might therefore be considered in symptomatic patients with an increase in femur or tibia torsion of at least 10° above normative values. Surgeons must be aware, however, that values strongly depend on the measurement technique used. In particular, the femur antetorsion can be assessed by multiple measurement techniques with normative values varying from 11° to 22° (Kaiser et al. 2016). Thus, for indicating a derotational osteotomy, values for femoral torsion always need to be interpreted relative to the measurement technique used.

### Scoring of Patellofemoral disorders

Throughout recent decades, the Kujala score has been the most widely used score for the evaluation of patellofemoral joint diseases. However, critical voices have been raised that doubt that this score is able to capture the complex nature of patellar instability and its associated problems (Tompkins and Arendt 2015). In particular, it is striking that patient cohorts achieved similar results regardless of whether they had experienced a patellar redislocation when the Kujala score was used as a clinical outcome measure (Balcarek et al. 2016; Erickson et al. 2015). To overcome this potential drawback, the implementation of more specifically designed patient-reported outcome measures for the assessment of patellar instability-related problems appeared necessary. For this purpose, two new assessment tools were developed: The Norwich Patellar Instability (NPI) score and the Banff Patella Instability Instrument (BPII) (Hiemstra et al. 2013; Lafave et al. 2016; Smith et al. 2014). Both scores are valid, reliable, and responsive for assessment of patients with patellar instability, and it is recommended to use at least one of these scores for future studies.

### Patellar instability in children and adolescents

#### Surgical treatment for this patient population is increasingly discussed

The incidence of patellar dislocation is highest among adolescents between 14 and 18 years, at 147.7 per 100.000 person-years (Sanders et al. 2017). Skeletally immature patients show redislocation rates of up to 71%, and younger age has been described as an outcome modifier (Lewallen et al. 2013; Lewallen et al. 2015). Jaquith and Parikh (2015) found a recurrence rate of about 43% in patients with open physis, whereas adolescents with closed physis had a recurrence rate of only 22% (Jaquith and Parikh 2015). Similarly, in a cohort study of children and adolescents, Lewallen et al. (2015) showed that patients with open physis had more than twice the risk of recurrent patellofemoral instability compared to patients with closed physis (Lewallen et al. 2015). Though the causal influence of age is not known, the high activity of children and adolescents might be the decisive factor. On the other hand, it can also be assumed that primary dislocation occurs sooner if risk factors are more pronounced. This is particularly evident by the fact that the simultaneous presence of a trochlear dysplasia and a patella alta increases the risk of redislocation to more than 70% (Jaquith and Parikh 2015).

The primary goal of treatment must be the reduction of recurrent patellofemoral instability episodes while allowing the affected children to return to their daily life and sport activities. The results of MPFL reconstruction are promising and superior to conservative treatment and to medial retinacular repair techniques alone (Bitar et al. 2012; Bitar et al. 2015). Therefore, surgical treatment for this population is increasingly discussed.

Several techniques for MPFL reconstruction in children and adolescents were described in recent literature. In contrast to adults, surgical procedures have to consider open physis and the potential risk of growth plate injury (Nelitz et al. 2013; Nelitz et al. 2017; Vavken et al. 2013). Therefore, most bony realignment techniques are contraindicated, and soft tissue procedures are designed to respect the anatomy. In this regard, the relationship of the femoral MPFL insertion to the medial femoral physis has been widely debated. Though some cadaveric studies have shown some more variability in the MPFL attachment at the femoral or patellar side in skeletally immature patients compared with adults (Shea et al. 2015; Shea et al. 2016), most studies found that the femoral origin of the MPFL was located distal to the physis (Nelitz et al. 2012; Shea et al. 2014; Kepler et al. 2011). This allows for an anatomic reconstruction using a femoral bone tunnel for MPFL graft fixation. However, during drilling into the distal femoral epiphysis at the MPFL origin in skeletally immature patients, the drill should be angled 15° to 20° both distally and anteriorly to minimize damage to the physis, notch, and distal femoral cartilage (Nguyen et al. 2017). Using this technique, Nelitz et al. (2017) reported the outcomes of twenty-five patients with a mean age of 12.8 years. All patients were treated with a pedicled quadriceps tendon flap for MPFL reconstruction and femoral bone tunnel fixation. After a mean of 2.6 years, no redislocation occurred, and 96% of the patients were very satisfied or satisfied with the result. In particular, no growth disturbance was noted (Nelitz et al. 2017). Parikh et al. (2013) used a similar technique and reported a redislocation rate of only 4.5% in a large cohort study of 179 MPFL reconstructions (Parikh et al. 2013). However, a 16.2% complication rate was noted. Those were related to recurrent patellar dislocation, patellar fracture, postoperative knee joint stiffness and anterior knee pain, but almost half of the complications resulted from technical problems.

‘Soft-tissue’ techniques of MPFL reconstruction for children and adolescents that avoid femoral tunnel drilling include the ‘adductor magnus tenodesis’, the ‘adductor sling’ technique and the semitendinosus or gracilis tendon tenodesis. The latter uses the medial collateral ligament as a pulley. The ‘adductor sling’ technique uses a free gracilis tendon that is looped around the adductor magnus tendon insertion. Lind et al. used the ‘adductor sling’ technique in a case series of twenty-four MPFL reconstructions in children aged 8–16 years (Lind et al. 2016). Though clinically relevant improvements were noted, 20% of the patients experienced a recurrent dislocation within the first postoperative year. The ‘adductor magnus tenodesis’ and the ‘modified adductor sling’ techniques yielded acceptable clinical results but showed increased rates of persistent instability, with rates of patellar redislocation of 10% and 13%, respectively (Alm et al. 2017; Malecki et al. 2015). Overall, several studies showed a lower risk of recurrent dislocations and good to excellent patient-reported outcome measures in the follow-up after surgical management of paediatric patellar instability (Nwachukwu et al. 2016; Nelitz et al. 2017; Lind et al. 2016; Wegmann et al. 2017). Though those studies were not directly comparative, a lower rate of patellar redislocation was found when an anatomic MPFL reconstruction with a femoral bone tunnel for MPFL graft fixation was used.

### Rehabilitation after patellar stabilization

The rehabilitation process after patellar stabilizing procedures often includes a knee immobilizer for 1–2 weeks and partial weight bearing (Asaeda et al. 2016; Krych et al. 2016). While some studies show range-of-motion restrictions for the first 2 weeks (Vitale et al. 2016), others allowed their patients free range of motion immediately postoperatively (Krych et al. 2016). To minimize the risk of arthrofibrosis, continuous passive or active motion should be applied within the first few days after surgery (Manske and Prohaska 2017). Cryotherapy and electrical stimulation are also used to decrease nerve conduction velocity and release endogenous opiates. Compression wraps can decrease existing swelling or prevent the onset of further swelling (Manske and Prohaska 2017). Full weight bearing and full range of motion should be possible 4–10 weeks after operative patella stabilization.

Isometric strengthening of the quadriceps muscle with straight-leg exercises is part of the early muscle activation process. Isolated training of the vastus medialis muscle is considered difficult and did not reach a clinically important difference when compared to a general quadriceps strengthening exercise programme. However, adductor muscle exercises also had the effect of recruiting the vastus medialis obliquus (VMO) muscle, and strengthening can therefore be achieved indirectly (Smith et al. 2010). Exercises are done 3 to 7 weeks after surgery (Vitale et al. 2016). Not only quadriceps exercises but also hip- and trunk-strengthening programmes are helpful and often mandatory to reduce the dynamic valgus instability of the lower extremity, which leads to lateral patellar maltracking and patellofemoral pain (Manske and Prohaska 2017; Petersen et al. 2017).

Isometric and isokinetic strengthening in closed-chain exercises should be adjusted to pain and range of motion (ROM) in each individual patient. Static-demand proprioceptive and neuromuscular control exercise programs should be done between 6 and 12 weeks postoperatively, with progression to dynamic proprioceptive training between 7 and 20 weeks postoperatively. Further progression to modified running programs can be allowed 10 to 21 weeks after MPFL surgery, and higher plyometric drills should be considered from 13 to 23 weeks postoperatively (Vitale et al. 2016).

To date, few studies have dealt with kinematic development after patellar stabilizing procedures. Asaeda et al. (2016) found that gait kinetics and gait kinematics had not been restored within 3 months after MPFL reconstruction but that parameters normalized within 1 year when compared to a healthy control group and to the preoperative status (Asaeda et al. 2016). Carnesecchi et al. (2016) showed a normal gait pattern in MPFL-reconstructed patients as early as 6 months after surgery while walking, but at higher speeds (>10 km/h), the single stance phase, the swing phase and the double-support phase were still significantly shortened (Carnesecchi et al. 2016).

Krych et al. evaluated the return to sport (RTS) rate. In their study of competitive athletes, 85% had an overall RTS rate after an average of 8.1 months following MPFL surgery. Patients with a concomitant tibial tubercle osteotomy had a longer return to sport time than those who underwent isolated MPFL (9.8 ± 5.5 vs. 7.0 ± 1.9 months) (Krych et al. 2016). In a study by Lippacher et al. (2014), 53% of patients were able to return to their original sports level or higher, whereas 47% performed at a lower level after MPFL reconstruction (Lippacher et al. 2014). In the lower sports level group, 10% of patients had a feeling of instability or had knee flexion deficits. Ménétrey et al. (2014) stated that patient education and regularly performed home exercises are other key factors that can lead to a successful return to sports (Ménétrey et al. 2014). Their criteria for a safe return to sports included absence of pain, no effusion, a complete range of motion, almost symmetrical strength, and excellent dynamic stability. Overall, it is becoming evident that clinical and functional test criteria are more important in the decision-making process of RTS than a simple time-based decision model (Zaman et al. 2017).

A ‘neuronal approach’ of rehabilitation was recently put forward by Kadowaki et al. (2017). Within their study group of MPFL-deficient knees, they found less activity in the contralateral somatosensory cortical areas, which may have led to diminished somatic sensation against lateral shifts of the patella (Kadowaki et al. 2017). In contrast, increased activity was found in the anterior cingulate cortex, prefrontal cortex and inferior parietal lobule. This activity may indicate anxiety of patellar instability and might be called a “neuronal positive apprehension sign”. Reconstruction of the MPFL influences surgically the stability of the patella, while neuromuscular training during rehabilitation may actively influence the “fear memory” of certain brain areas in surgically reconstructed knees. Future rehabilitation programs should therefore focus not only on peripheral neuromuscular function but also on neurocognitive training to re-educate the central nervous system (Kadowaki et al. 2017).
